# Aliskiren Effect on Plaque Progression in Established Atherosclerosis Using High Resolution 3D MRI (ALPINE): A Double-Blind Placebo-Controlled Trial

**DOI:** 10.1161/JAHA.112.004879

**Published:** 2013-06-21

**Authors:** Georgeta Mihai, Juliet Varghese, Thomas Kampfrath, Liubov Gushchina, Lisa Hafer, Jeffrey Deiuliis, Andrei Maiseyeu, Orlando P. Simonetti, Bo Lu, Sanjay Rajagopalan

**Affiliations:** The Dorothy M. Davis Heart & Lung Research Institute and the Division of Cardiovascular Medicine, The Ohio State University, Columbus, OH (G.M., J.V., T.K., L.G., L.H., J.D., A.M., O.S., S.R.); College of Public Health, The Ohio State University, Columbus, OH (B.L.)

**Keywords:** atherosclerosis, magnetic resonance imaging, renin

## Abstract

**Background:**

The renin–angiotensin system is well recognized as a mediator of pathophysiological events in atherosclerosis. The benefits of renin inhibition in atherosclerosis, especially when used in combination with angiotensin-converting enzyme inhibitors/angiotensin receptor blockers (ACEIs/ARBs) are currently not known. We hypothesized that treatment with the renin inhibitor aliskiren in patients with established cardiovascular disease will prevent the progression of atherosclerosis as determined by high-resolution magnetic resonance imaging (MRI) measurements of arterial wall volume in the thoracic and abdominal aortas of high-risk patients with preexisting cardiovascular disease.

**Methods and Results:**

This was a single-center, randomized, double-blind, placebo-controlled trial in patients with established cardiovascular disease. After a 2-week single-blind placebo phase, patients were randomized to receive either placebo (n=37, mean±SD age 64.5±8.9 years, 3 women) or 150 mg of aliskiren (n=34, mean±SD age 63.9±11.5 years, 9 women). Treatment dose was escalated to 300 mg at 2 weeks and maintained during the remainder of the study. Patients underwent dark-blood, 3-dimensional MRI assessment of atherosclerotic plaque in the thoracic and abdominal segments at baseline and on study completion or termination (up to 36 weeks of drug or matching placebo). Aliskiren use resulted in significant progression of aortic wall volume (normalized total wall volume 5.31±6.57 vs 0.15±4.39 mm^3^, *P*=0.03, and percentage wall volume 3.37±2.96% vs 0.97±2.02%, *P*=0.04) compared with placebo. In a subgroup analysis of subjects receiving ACEI/ARB therapy, atherosclerosis progression was observed only in the aliskiren group, not in the placebo group.

**Conclusions:**

MRI quantification of atheroma plaque burden demonstrated that aliskiren use in patients with preexisting cardiovascular disease resulted in an unexpected increase in aortic atherosclerosis compared with placebo. Although preliminary, these results may have implications for the use of renin inhibition as a therapeutic strategy in patients with cardiovascular disease, especially in those receiving ACEI/ARB therapy.

**Clinical Trial Registration:**

URL: http://ClinicalTrials.gov Unique identifier: NCT01417104.

## Introduction

Epidemiological and experimental data suggest that activation of the renin–angiotensin system (RAS) plays an important role in the pathogenesis of atherosclerosis.^1,2^ Studies in patients with established atherosclerosis or who are at high risk for cardiovascular events have demonstrated favorable effects of RAS antagonists such as angiotensin-converting enzyme inhibitors (ACEIs) or angiotensin receptor blockers (ARBs).^3,4^ It has been hypothesized that proximal inhibition of renin, a rate-limiting step that mediates the conversion of angiotensinogen to angiotensin I, may exert more complete inhibition of the RAS cascade, leading to the prevention of recurrent events.^5,6^ In keeping with this concept, renin inhibition reduces atherosclerotic plaque progression in both murine and rabbit models.^7–9^ However, clinical evidence from contemporary randomized controlled clinical trials of ACEI/ARB therapy in patients at high risk for vascular disease and/or with established disease has been neutral and in some cases appears to suggest a paradoxical increase in cardiovascular adverse events, especially when used in combination.^10–12^ Given the current uncertainty regarding the clinical use of renin inhibition for the prevention of cardiovascular events, we investigated the effect of a renin inhibitor (aliskiren) in aortic plaque progression in a high-risk patient population with established atherosclerosis using high-resolution, 3-dimensional magnetic resonance imaging (MRI) quantification of aortic wall volume. By directly measuring the effect on the vessel wall of cumulative cardiovascular risk factors, plaque progression/stability assessment with the use of MRI has been demonstrated to represent a biomarker for disease and to assess therapeutic effects.^13^ In this single-center experience, the use of a renin inhibitor resulted in plaque progression in a high-risk normotensive patient population, most of whom were already receiving ACEI/ARB therapy.

## Methods

### Patients and Study Design

The ALiskiren effect on Plaque progression IN Established atherosclerosis using high resolution 3D MRI (ALPINE) trial was designed as a prospective, single-center, double-blind, placebo-controlled, parallel-group study. The Ohio State University institutional review board approved the protocol, and all patients provided written consent. The study's primary end point was the difference between the placebo and aliskiren groups in change in the normalized total aortic wall volume (TWV) at the end of the treatment period. Aortic wall volume was quantified in the thoracic and abdominal aortas (extending from the origin of the subclavian to the renal arteries) with the use of high-resolution, 3-dimensional, dark-blood MRI. The secondary end point was the difference between the aliskiren and placebo groups in change in the percentage wall volume (PWV) in the aorta between baseline and the end of the treatment period. Tertiary measures included (1) the change in resting clinic systolic blood pressure (SBP) at the end of the treatment period, (2) the change in central aortic pressure measured with applanation tonometery (SphygmoCor CP, AtCor Medical, Itaska, Ill), and (3) changes in plasma measures of inflammation, insulin resistance, and adipokines.

#### Inclusion criteria

Patients, both men and women, were eligible if they were ≥45 years of age, with previously documented cardiovascular disease, defined as at least 1 of the following: myocardial infarction, cerebrovascular accident, previous coronary artery bypass graft surgery, and/or percutaneous coronary intervention (PIC) or peripheral arterial disease, defined as ankle-brachial index <0.9 and/or prior peripheral intervention or surgery. Subjects receiving ACEI or ARB therapy were eligible to participate, provided no dose adjustments were made during the course of the study.

#### Exclusion criteria

Exclusion criteria were contraindications to the MRI examination (eg, pacemakers, metallic implants, severe claustrophobia); diagnosis of type 1 diabetes or use of hypoglycemic drugs; uncontrolled hypertension (>145/90 mm Hg); low-density lipoprotein (LDL) of ≥130 mg/dL; renal insufficiency defined as glomerular filtration rate ≤40 mL/min (derived with the Modified Diet in Renal Disease equation); initiation of new therapy with statins, ACEIs/ARBs, antioxidants, calcium channel blockers, diuretics, or β-blockers; transient ischemic cerebral attack during the prior 6 months; history of allergy to renin inhibitors; unstable cardiac syndromes; symptomatic arrhythmias; history of malignancy including leukemia and lymphoma (but not basal cell skin cancer, cured squamous cell cancer, or localized prostate cancer); and history of allergy to renin inhibitors.

### Treatment Blinding and Randomization Scheme

Both patients and study personnel were blinded to treatment assignment. Randomization was performed using computer-generated random numbers at the Division of Biostatistics, College of Public Health, Ohio State University. Enrolled subjects meeting inclusion criteria were randomized 1:1 to placebo or aliskiren. At randomization, patients were stratified by ACEI/ARB use or nonuse.

### Treatments and Clinic Visits

**Figure 1.**
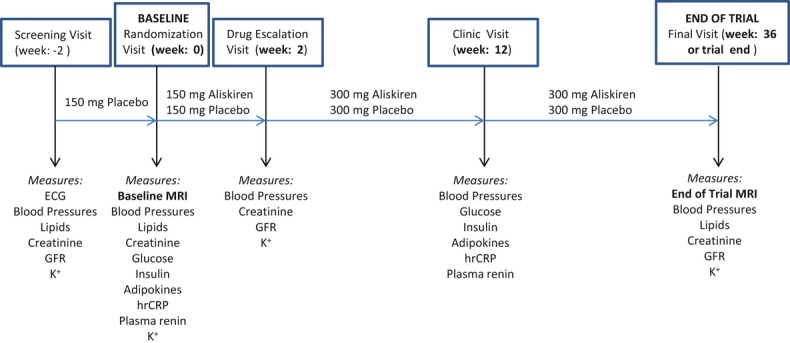
Trial timeline. GFR indicates glomerular filtration rate; CRP, C-reactive protein.

The 36-week double-blind, randomized-treatment phase of the trial was preceded by a 2-week single-blind placebo period to assess eligibility for the active treatment period, to assess compliance, and to confirm the baseline blood pressure values of the enrolled subjects. If inclusion criteria were met at the end of the single-blind phase, the participants were allowed to proceed to the trial and undergo baseline MRI studies after being randomized to either placebo or aliskiren 150 mg, with an escalation to 300 mg at 2 weeks into treatment. This dose was then maintained for the duration of the trial. After randomization and dose-escalation visits (at 2 weeks), patients returned for scheduled clinic visits at weeks 12 and 36 (or end of trial). Assessment of routine safety measures including serum creatinine and potassium levels was performed at predesignated visits (randomization, drug escalation, and end of trial). At each study visit, after having the patient in a sitting position for 5 minutes, SBP/diastolic blood pressure was measured 3 times in accordance with the American Heart Association Council on High Blood Pressure Research Professional and Public Education Subcommittee report on blood pressure determination.^14^ The patient was then asked to stand for 2 minutes, and a single blood pressure measurement was measured in the standing position. Evidence of left ventricular hypertrophy was determined using the Romhilt-Estes scoring system at baseline.^15^ Specialized measurements of plasma, including insulin, glucose, adipokines (leptin and adiponectin), and high-sensitivity C-reactive protein, were performed at randomization and 12 weeks into the trial, at a core laboratory blinded to treatment assignment (Michigan Diabetes Research and Training Center, University of Michigan, Ann Arbor, Mich). Central aortic blood pressure assessment was performed at randomization and end-of-trial/exit visits (SphygmoCor CP, AtCor Medical). Plasma direct renin measurements were obtained at baseline and 12 weeks, in part to assess compliance of patients with their therapy (Diasource, Louvain-La-Neuve, Belgium). A summary of the study timeline and data collection is presented in Figure 1.

On December 21, 2011, the ALPINE investigators received notification from the sponsors about termination of the study based on results of the Aliskiren Trial In Type 2 Diabetes Using Cardio-Renal Endpoints (ALTITUDE) (http://clinicaltrials.gov/ct2/show/NCT00853827) that raised safety concerns regarding the use of aliskiren by diabetic patients receiving ACEI/ARB therapy. Patients were notified and brought back for the end-of-trial visit, which included the MRI study provided they had undergone at least 17 weeks of therapy on the trial.

### Aortic MRI

All patients underwent MRI with a 3-T, 32-channel, whole-body MRI system (either Tim Trio or Verio, Siemens, Erlangen, Germany) with maximum gradient amplitude of 45 mT/m and a slew rate of 200 mT/m per millisecond. The system's integrated body coil was used for radiofrequency signal transmission, and 2 flexible 6-element phase-array body coils were used for MR signal reception. After localization, an ECG-triggered and respiratory-navigated acquisition with an acceptance window of ±2.5 mm was used to image the aorta. The MRI sequence method used for wall depiction was a 3-dimensional, fat-suppressed, dark-blood, turbo spin-echo sequence with variable flip angles (Sampling Perfection with Application-optimized Contrast with different flip angle Evolutions [SPACE]), which was previously described by our group.^16,17^ The complete details of the MRI acquisition are provided in Table 1.

**Table 1.** MRI Acquisition Parameters

Parameters	3D-T1-Weighted SPACE
Resolution, mm^3^	1.1×1.1×1.1

Navigator gated	Yes

ECG gated	Yes

TR/TE, ms	R-R/21

Slab orientation	Sagittal/oblique

FOV, cm^2^	320×271

Averages	1.4

Slices per slab	64

Matrix size	304/246

BW, Hz/Px	783

Echo spacing, ms	3.4

Turbo factor	45

Slice turbo factor	1

Echo train per slice	3

Echo train duration, ms	156

MRI indicates magnetic resonance imaging; SPACE, sampling perfection with application-optimized contrast with different flip angle evolutions; TR, repetition time; TE, echo time; FOV, field of view; BW, bandwidth.

### Image Analysis

Image analysis was performed in a blinded manner by a trained observer with no information on the patients or randomization status. Before analysis, the images were recalled onto a workstation (Leonardo, Siemens). The MR images were visually assessed for overall image quality and vessel wall delineation. The 3-dimensional data sets from the initial and subsequent studies were coregistered using 3-dimensional postprocessing software (FUSION, Siemens Healthcare Inc, Erlangen, Germany). Multiplanar reconstructed consecutive images, perpendicular to the aorta, were generated at the time of coregistration, from both data sets at the same matched location. Thoracic multiplanar reconstructed cross-sectional images were generated from cuts perpendicular to the aorta, starting after the level of the left subclavian artery origin and stopping at the diaphragm level. Abdominal transverse cuts were generated on aorta spanning from the diaphragm to the level of the renal arteries. After coregistration and the generation of multiplanar reconstructed sections, images were magnified, image contrast was adjusted, and patient/examination identifier information was removed and replaced by a preassigned code to blind images for measurements.

**Figure 2.**
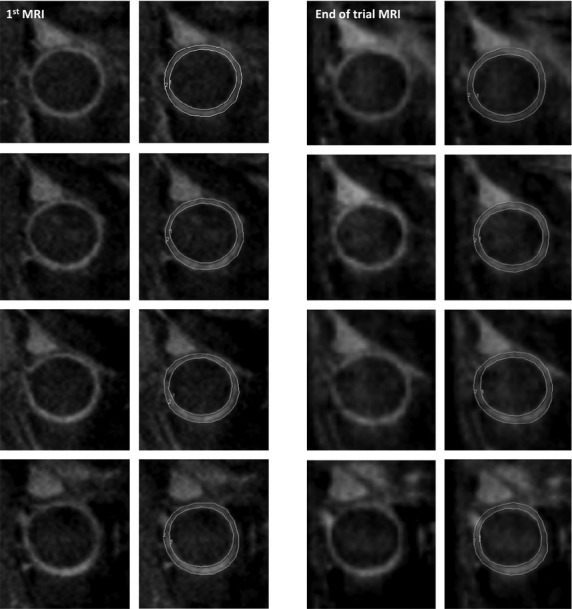
Thoracic aorta multiplanar reconstructed (MPR) cuts without (left) and with (right) arterial wall delineation contours, showing matched magnetic resonance imaging (MRI) slices (1.1 mm thick) of the baseline MRI (left group) and end of trial MRI (right group) examinations in a 62-year-old man.

**Figure 3.**
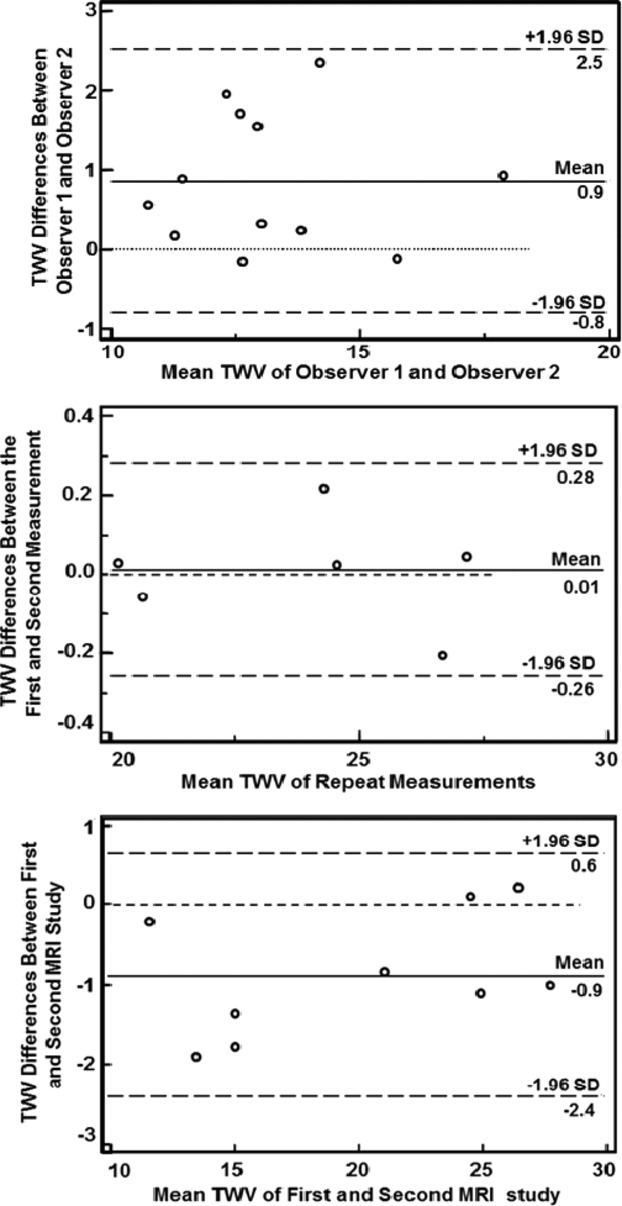
Bland–Altman plots showing the interobserver (top), intraobserver (middle), and interscan (bottom) variability for the thoracic aorta total normalized wall volume (TWV). MRI indicates magnetic resonance imaging.

An experienced observer blinded to the data's old header information performed manual measurements of lumen and lumen-plus-wall areas by delineating the inner border and the outer border of the vessel wall in each cross-sectional image of the aorta (Figure 2). Vessel wall area (WA) for each patient was calculated by subtracting lumen area (LA) from the outer border wall area. Using an approach similar to intravascular atheroma volume calculations, the secondary efficacy parameter, PWV, was calculated as:


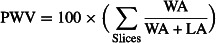
(1)

for thoracic region, abdominal region, and total aorta for each patient and each examination. The mean wall area (MWA) was calculated for each patient as:


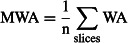
(2)

where n is the number of matched cross sections of aorta at baseline and the end of the study. The primary parameter—the normalized TWV—was calculated as:


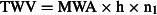
(3)

where h is the MRI slice thickness (1.1 mm) and n_1_ is the median number of matched cross-sectional cuts for all patients. After completion of the wall measurement process, data were unblinded and changes in PWV and TWV were determined for each patient for thoracic and abdominal aortic segments, as well as for the entire aorta if they had measurements on both aortic segments. Of the 37 patients who had baseline and end-of-trial MRI examinations, 34 had cross-sectional segments and measurements of the thoracic aorta (median [interquartile range (IQR)]: 100 [80 to 100]), 31 had multiplanar reconstructed images in the abdominal aorta (median [IQR]: 90 [80 to 100]), and 28 had both thoracic and abdominal images for wall measurements (median [IQR]: 182.5 [160 to 200]). Reproducibility measurements to assess the interobserver and intraobserver agreements for wall volume measurements were performed in 12 patient data sets (n=50 slices per patient) for the interobserver reproducibility and in 6 patient data sets (n=150 slices per patient) for the intraobserver agreement. Also, scan repeatability (mean duration between repeat scans 8.8±7.2 days) was assessed in 5 patient and 4 normal subject data sets (n=150 slices per subjects) by 1 observer. The intracorrelation coefficient for all reproducibility (intraobserver and interobserver), as well as the scan–rescan repeatability measurements, was >0.85. Bland–Altman plots depicting levels of agreement between 2 independent reviewer measurements, within-observer repeat measurements and between-observer repeat scans as assessed by 1 observer, are shown in Figure 3 for TWA at the thoracic aorta level.

### Statistical Methods

We estimated that a sample size of 28 patients in each group will have 80% power to detect a difference of 0.25 cm^3^ in total plaque volume between the placebo and treatment groups at a significance level of 0.05. This assumed an SD of 0.35 cm^3^, which was based on preliminary studies and on prior studies that have reported changes in thoracic and abdominal aortic plaque with statin therapy.^18^ Assuming a dropout rate of 20%, enrollment of 35 patients per treatment group was specified to provide an adequate number of evaluable MRI patients. For primary and secondary analyses, changes in measures were calculated as differences between measures taken at follow-up examination and at baseline. Continuous variables are presented as mean with SD and 95% confidence interval for the mean, median, and IQR values and compared with use of Wilcoxon rank sum test. Categorical variables are presented as proportion and compared with use of the Pearson χ^2^ test. Linear regression analyses were conducted to identify important predictors (age, gender, baseline SBP, total and LDL cholesterol, and treatment duration) of the primary outcomes. All statistical tests were 2-sided. A *P* value <0.05 was considered significant for all analyses. All statistical analyses were performed using Stata version 12.0 (StataCorp LP. College Station, Tex).

## Results

### Patient Population

**Figure 4.**
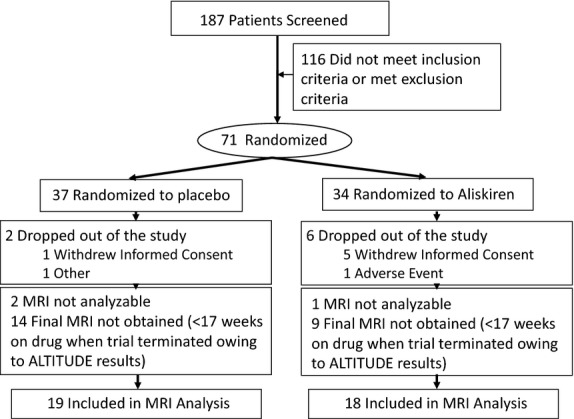
Flow of patients through the trial.

Between April 2010 and December 2011, 187 patients were screened from the patient population at The Ohio State Wexner Medical Center and Columbus area. The flow of patients through the trial is presented in Figure 4. A total of 71 patients who meet all the inclusion criteria for study participation were randomized (placebo=37 and aliskiren=34 patients). Two patients from the placebo group and 6 patients from the aliskiren group dropped out from the trial in the titration phase owing to laboratory abnormalities (hyperkalemia, n=1) or withdrawal of consent, leaving (as of December 2011) 35 patients in the placebo group and 28 patients in the aliskiren group to complete the study. After the study was terminated by the sponsor in December 2011 because of the results in the ALTITUDE trial, as mentioned earlier, patients were asked to stop the study drug and were required to have a termination visit that included a repeat MRI examination. The main results of the ALTITUDE trial demonstrated a 7% increase in major adverse cardiovascular and renal events in the aliskiren arm, which was primarily driven by a 25% increase in stroke rate and resuscitated sudden death. Baseline demographic data for the 71 randomized patients are reported in Table 2. There were no differences in baseline characteristics in patients randomized to the 2 treatment arms, except that a greater number of women were enrolled in the aliskiren arm. On average, patients were >60 years old, and the majority were obese (body mass index >30 kg/m^2^) with documented prior vascular disease. Approximately 60% of the placebo group patients and 61% of the aliskiren group patients were receiving ACEI/ARB therapy. A total of 27 patients had completed the trial and 36 patients were actively enrolled in the study at the time of the decision to end the trial. Of the 36 patients, 7 patients were enrolled in the trial for 30 to 37 weeks, 6 patients were enrolled for 19 to 29 weeks, and 23 were enrolled for <19 weeks. Patients who completed at least 19 weeks in the trial (receiving study medication for 17 weeks) underwent an end-of study MRI examination. All subjects included in the study analysis reported >90% drug compliance during the study.

**Table 2.** Baseline Characteristics of the Patient Population

Parameters	Placebo (n=37)	Aliskiren (n=34)
Age, mean y (SD)	64.5 (8.9)	63.9 (11.5)

Body mass index, mean kg/m^2^ (SD)	29.4 (4.5)	30.8 (5.3)

Total cholesterol, mean mg/dL (SD)	156.9 (27.2)	169.4 (36.4)

Gender (male), n (%)*	34 (91.9)	25 (73.5)

GFR, mean (SD)	59.8 (0.73)	57.8 (5.7)

ACE/ARB, n (%)	22 (59.5)	21 (61.8)

Statin treatment, n (%)	24 (64.9)	28 (82.4)

ASA and/or ADP receptor antagonists, n (%)	30 (81.1)	27 (79.4)

β-Blockers, n (%)	25 (67.6)	24 (70.6)

Calcium channel blockers, n (%)	3 (8.1)	4 (11.8)

Direct vasodilators, n (%)	0 (0)	1 (2.9)

Aldosterone receptor antagonists, n (%)	0 (0)	0 (0)

HCTZ, n (%)	4 (10.8)	4 (11.8)

Smoking, n (%)		

Never	33 (89.2)	23 (67.7)

Former	2 (5.4)	3 (8.8)

Present	2 (5.4)	8 (23.5)

Race, n (%)		

White	33 (89.2)	31 (91.2)

African-American	3 (8.1)	3 (8.8)

Asian	1 (2.7)	0 (0)

Evidence of LVH (ECG), n (%)	19 (51.4)	16 (47.1)

Clinical target organ damage, n (%)		

MI	12 (32.4)	19 (55.9)

CAD (stents)	29 (78.4)	18 (52.9)

CAD (CABG)	10 (27.0)	11 (32.4)

CVA	3 (8.1)	3 (8.8)

PAD	8 (21.6)	8 (23.5)

Multiple organ beds (≥2)	17 (45.9%)	18 (52.9%)

ASA indicates acetylsalicylic acid; GFR, glomerular filtration rate; HCTZ, hydrochlorothiazide; LVH, left ventricular hypertrophy; MI, myocardial infarction; CAD, coronary artery disease, CABG, coronary artery bypass graft surgery; CVA, cerebrovascular accident; PAD, peripheral artery disease.

* GFR units are mL/min/1.73 m^2^. 0.01<*P* value<0.05.

### Blood Pressure Measurements and Laboratory Assessment

Baseline sitting clinic SBP was 126.87±12.54 mm Hg in placebo group patients and 124.86±17.85 mm Hg in aliskiren group patients, respectively. Baseline average values of plasma renin concentration for the placebo and aliskiren groups were 35.5±69.5 pg/mL and 33.6±54.5 pg/mL, respectively, with a significant increase at 12 weeks for the aliskiren group to 327.9±526 pg/mL, whereas renin levels in the placebo group remained unchanged at 38.8±85.3 pg/mL. Table 3 lists blood pressure changes during the trial. There were no significant differences between the 2 groups in any of the hemodynamic measures including central aortic pressures. Table 4 summarizes the lipid, insulin, glucose, adipokine (leptin, adiponectin), and high-sensitivity C-reactive protein measurements during the trial. There were no significant differences between the 2 study arms at baseline in any of the lipid or metabolic measures. There was a significant difference (*P*<0.05) in leptin changes in the aliskiren group compared to the placebo group. At follow-up, C-reactive protein measures were similar for the placebo and aliskiren groups.

**Table 3.** Blood Pressure Changes During Treatment

	Placebo		Aliskiren	
		
	Mean (SD)	[95% CI]	Mean (SD)	[95% CI]
Baseline examination (visit 1/visit 2)				

Sitting SBP*	126.87 (12.54)	[122.56 to 131.18]	124.86 (17.85)	[117.94 to 131.78]

Sitting DBP*	78.16 (8.71)	[75.17 to 81.16]	77.37 (6.93)	[74.68 to 80.06]

Standing SBP*	126.03 (15.76)	[120.62 to 131.44]	123.89 (19.95)	[116.16 to 131.63]

Standing DBP*	79.74 (10.37)	[76.18 to 83.31]	79.46 (9.22)	[75.89 to 83.04]

Sitting MAP*	94.40 (8.86)	[91.35 to 97.44]	93.20 (9.54)	[89.5 to 96.9]

Central SBP6	120.58 (10.5)	[116.86 to 124.3]	118.64 (17.31)	[111.50 to 125.78]

Central DBP†	79.55 (8.76)	[76.44 to 82.65]	78.04 (8.24)	[74.64 to 81.44]

Follow-up examination (end of trial)				

Sitting SBP*	124.54 (9.79)	[121.18 to 127.91]	120.98 (10.8)	[116.79 to 125.16]

Sitting DBP*	76.99 (7.01)	[74.58 to 79.4]	75.05 (5.93)	[72.75 to 77.35]

Standing SBP*	126.46 (15.25)	[121.22 to 131.7]	121.0 (13.18)	[115.89 to 126.11]

Standing DBP*	78.17 (7.77)	[75.5 to 80.84]	75.57 (7.67)	[72.6 to 78.54]

Sitting MAP*	92.84 (6.97)	[90.45 to 95.23]	90.36 (6.75)	[87.74 to 92.98]

Central SBP†	117.49 (10.51)	[113.76 to 121.21]	117.00 (11.95)	[112.07 to 121.93]

Central DBP†	78.52 (7.91)	[75.71 to 81.32]	77.72 (6.61)	[74.99 to 80.45]

Change from baseline				

Sitting SBP*	−2.32 (11.83)	[−6.39 to 1.74]	−3.88 (13.8)	[−9.23 to 1.47]

Sitting DBP*	−1.17 (6.89)	[−3.54 to 1.2]	−2.32 (7.9)	[−5.39 to 0.74]

Standing SBP*	0.43 (19.89)	[−6.41 to 7.26]	−2.89 (19.31)	[−10.38 to 4.6]

Standing DBP*	−1.57 (7.52)	[−4.15 to 1.01]	−3.89 (9.47)	[−7.56 to −0.22]

Sitting MAP*	−1.56 (7.6)	[−4.17 to 1.06]	−2.84 (8.83)	[−6.26 to 0.58]

Central SBP†	−3.09 (12.64)	[−7.57 to 1.39]	−1.64 (14.7)	[−7.71 to 4.43]

Central DBP†	−1.03 (6.85)	[−3.46 to 1.4]	−0.32 (8.91)	[−3.99 to −3.36]

All values given in mm Hg; SBP indicates systolic blood pressure; DBP, diastolic blood pressure; MAP, mean arterial pressure.

* Placebo (n=35), aliskiren (n=28).

† Baseline values acquired at visit 2. Placebo (n=33), aliskiren (n=25).

**Table 4.** Lipid, Metabolic, and Adipokine Changes During Treatment

	N	Baseline (Visit 2) Mean (SD), [95% CI]	Follow-Up (Visit 4/End of Trial) Mean (SD), [95% CI]	Change Mean (SD), [95% CI]
Total cholesterol*, mg/dL				

Placebo	33	176.67 (61.06), [155.02 to 198.32]	160.61 (42.15), [145.66 to 175.55]	−16.06 (77.32), [−43.48 to 11.36]

Aliskiren	27	166.96 (28.76), [155.59 to 178.34]	157.93 (25.33), [147.91 to 167.95]	−9.04 (42.36), [−25.79 to 7.72]

HDL cholesterol*, mg/dL				

Placebo	33	43.94 (13.82), [39.04 to 48.84]	41.85 (9.97), [38.31 to 45.38]	−2.09 (9.59), [−5.49 to 1.31]

Aliskiren	27	41.59 (10.06), [37.61 to 45.57]	42.96 (10.05), [38.99 to 46.94]	1.37 (6.81), [−1.32 to 4.07]

Non-HDL cholesterol*, mg/dL				

Placebo	33	132.73 (58.50), [111.98 to 153.47]	118.76 (40.91), [104.25 to 133.26]	−13.97 (74.09), [−40.24 to 12.3]

Aliskiren	27	125.37 (31.07), [113.08 to 137.66]	114.96 (28.18), [103.82 to 126.11]	−10.41 (45.17), [−28.28 to 7.46]

Glucose, mg/dL				

Placebo	26	110.31 (32.03), [97.37 to 123.25]	115.81 (26.19), [105.23 to 126.39]	5.5 (22.88), [−3.74 to 14.74]

Aliskiren	23	106.35 (20.88), [97.32 to 115.38]	107.83 (17.02), [100.47 to 115.19]	1.48 (23.8), [−8.81 to 11.77]

Insulin, μU/mL				

Placebo	26	25.75 (30.16), [13.57 to 37.93]	41.27 (78.5), [9.56 to 72.98]	15.52 (50.94), [−5.06 to 36.1]

Aliskiren	23	38.83 (38.57), [22.15 to 55.51]	32.68 (17.91), [24.94 to 40.43]	−6.15 (34.18), [−20.93 to 8.63]

HOMA-IR				

Placebo	26	7.75 (11.84), [2.96 to 12.53]	14.83 (37.7), [−0.4 to 30.05]	7.08 (26.52), [−3.63 to 17.8]

Aliskiren	23	11.10 (12.78), [5.57 to 16.62]	9.00 (5.91), [6.45 to 11.56]	−2.1 (11.68), [−7.15 to 2.95]

Leptin†, ng/mL				

Placebo	26	12.15 (14.96), [6.11 to 18.20]	11.29 (10.69), [6.97 to 15.6]	−0.87 (5.55), [−3.11 to 1.38]

Aliskiren	23	21.63 (19.51), [13.19 to 30.06]	23.87 (21.37), [14.62 to 33.11]	2.24 (5.09), [0.04 to 4.44]

Adiponectin, ng/mL				

Placebo	26	5670.74 (3812.03), [4131.03 to 7210.46]	5970.70 (4101.71), [4313.99 to 7627.42]	299.95 (1000.87), [−104.31 to 704.21]

Aliskiren	22	7298.55 (5203.32), [4991.52 to 9605.57]	7595.15 (6032.19), [4920.62 to 10 269.67]	296.55 (2372.72), [−755.46 to 1348.55]

hsCRP, mg/L				

Placebo	26	1.69 (3.92), [0.11 to 3.27]	2.12 (4.61), [0.26 to 3.98]	0.43 (1.55), [−0.2 to 1.06]

Aliskiren	23	4.31 (5.22), [2.05 to 6.57]	4.22 (5.76), [1.73 to 6.71]	−0.09 (3.66), [−1.68 to 1.49]

HDL indicates high-density lipoprotien; HOMA-IR, Homeostasis Model Assessment–Insulin Resistance; hsCRP, high-sensitivity C-reactive protein.

* Follow-up is end of trial for lipid measures.

† 0.01<*P* value<0.05.

### MRI Wall Volume Measurements

**Figure 5.**
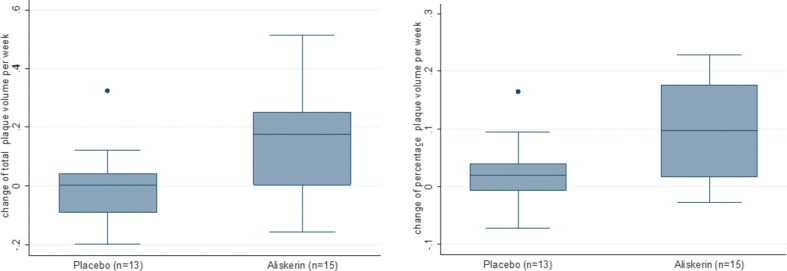
Weekly MRI measures changes (TWV, left; PWV, right) shown for the total aorta for the 2 treatment groups. There is a statistical significant difference (*P*<0.05) between the treatment arms. PWV indicates percentage wall volume; TWV, total normalized wall volume.

Table 5 provides the results for the primary (TWV) and secondary (PWV) MRI measures and enumerates the values for total aorta and thoracic and abdominal aortic segments at baseline and follow-up and for changes during the study. Baseline MRI outcomes showed a significant difference between the 2 treatment arms for the abdominal and thoracic TWV, with the placebo group demonstrating higher baseline plaque values compared with the aliskiren group (25.37±4.96 vs 22.43±3.81 cm^3^ for thoracic TWV and 22.76±4.64 vs 19.11±4.07 cm^3^ for abdominal TWV, *P*=0.04 for placebo vs aliskiren for both measures, respectively; Table 5). During the study period (mean drug exposure duration of 25 weeks), both the placebo and the aliskiren group demonstrated progression with regard to their respective baseline plaque measures. The primary end point of the study—mean change in normalized TWV between the 2 treatment groups—was significantly higher for the aliskiren group (mean difference between aliskerin and placebo groups 4.16 cm^3^, *P*=0.03, aliskiren vs placebo). The secondary MRI end point in the study—change in PWV between aliskiren and placebo—was also significantly different (3.41% higher in aliskiren group, *P*=0.02, aliskiren vs placebo). When comparing the progression of plaque between the aliskiren and placebo groups, there were interesting differential changes noted in the thoracic and abdominal segments. The progression rates of MRI measures in the abdominal segments were no different when comparing baseline with end-of-study measures (TWV and PWV) for both groups. In contrast, the thoracic segments demonstrated significant progression in wall volume by both TWV and PWV between baseline and end-of-study measures in both treatment groups. There was no effect of age, gender, baseline SBP, or total or LDL cholesterol on plaque measures in either group. Linear regression analysis to assess the effect of duration of treatment on plaque progression revealed that the number of weeks receiving aliskiren was a significant predictor for change in both TWV and PWV MRI measures. The changes per week in total aortic TWV and PWV are depicted in Figure 5. For aliskiren compared with placebo, remaining on medication 1 week longer resulted in an average increase of 0.43 cm^3^ in TWV and of 0.23% in PWV.

**Table 5.** Quantitative MRI Aortic Wall Measures (TWV and PWV) at Baseline and Follow-up and Changes From Follow-up in Patients Who Have Completed the Trial With Baseline and End-of-Trial MRI Examinations (n=37)

	Placebo (n=19)		Aliskiren (n=18)		
			
	Mean (SD)	Median (IQR)	Mean (SD)	Median (IQR)	*P*
Baseline examination					

TWV total*	45.84 (8.94)	46.91 (41.00 to 50.73)	39.86 (7.44)	37.5 (35.73 to 44.3)	0.08

TWV thoracic†	25.37 (4.96)	25.44 (22.95 to 28.26)	22.43 (3.81)	22.1 (20.3 to 24.45)	**0.04**

TWV abdominal‡	22.76 (4.64)	22.53 (19.36 to 26.34)	19.11 (4.07)	17.86 (16.48 to 21.02)	**0.04**

PWV total*	33.10 (2.34)	32.52 (31.48 to 33.89)	33.9 (1.96)	34.16 (32.67 to 34.8)	0.19

PWV thoracic†	30.86 (3.09)	29.54 (28.88 to 33.4)	32.64 (2.38)	32.9 (31.24 to 34.59)	0.08

PWV abdominal‡	34.66 (2.58)	34.04 (32.66 to 37.12)	35.29 (2.43)	35.08 (33.32 to 36.75)	0.53

Follow-up examination					

TWV total*	45.99 (8.96)	47.30 (38.84 to 51.7)	45.17 (11.18)	44.15 (37.07 to 48.65)	0.48

TWV thoracic†	28.88 (5.57)	29.25 (24.92 to 39.93)	27.71 (6.08)	27.01 (22.55 to 31.16)	0.3

TWV abdominal‡	21.41 (5.55)	20.57 (17.07 to 24.15)	19.03 (5.53)	17.96 (15.33 to 19.33)	0.11

PWV total*	34.05 (3.09)	32.81 (31.80 to 37.53)	37.27 (2.91)	37.75 (35.56 to 39.77)	**0.02**

PWV thoracic†	34.69 (4.32)	33.76 (32.37 to 37.90)	37.94 (2.61)	37.79 (36.62 to 39.52)	**0.016**

PWV abdominal‡	33.83 (4.35)	33.06 (30.21 to 36.76)	35.90 (4.39)	36.91 (32.64 to 38.57)	0.11

	Mean (SD) [95% CI]	*P value* (within group)	Mean (SD) [95% CI]	*P value* (within group)	*P* value (across group)
Changes between baseline and follow-up					

TWV total*	0.15 (4.39) [−2.51 to 2.8]	0.70	5.31 (6.57) [1.67 to 8.95]	**0.01**	**0.03**

TWV thoracic†	3.51 (2.43) [2.22 to 4.81]	**<0.001**	5.28 (3.06) [3.76 to 6.8]	**<0.001**	0.08

TWV abdominal‡	−1.35 (5.03) [−4.03 to 1.33]	0.12	−0.08 (4.24) [−2.43 to 2.26]	0.99	0.25

PWV total*	0.97 (2.02) [−0.25 to 2.19]	0.09	3.37 (2.96) [1.73 to 5.01]	**0.003**	**0.04**

PWV thoracic†	3.83 (2.35) [2.58 to 5.10]	**<0.001**	5.3 (2.2) [4.2 to 6.39]	**<0.001**	0.13

PWV abdominal‡	−0.83 (4.09) [−3.01 to 1.35]	0.25	0.61 (4.17) [−1.7 to 2.92]	0.57	0.29

PWV indicates percentage wall volume; TWV, total normalized wall volume.

* Placebo (n=13), aliskiren (n=15).

† Placebo (n=16), aliskiren (n=18).

‡ Placebo (n=16), aliskiren (n=15).

To evaluate the effect of ACEI/ARB therapy on changes in the MRI end-points, we compared the progression in TWV and PWV between the placebo and aliskiren groups according to ACEI/ARB use (Table 6). Only for patients receiving ACEIs/ARBs, the rate of progression as measured by both TWV and PWV was significantly higher in the aliskiren group compared with the placebo group for both TWV and PWV (*P*=0.016, mean 7.53 cm^3^, median 8.2 cm^3^ for ΔTWV; *P*=0.027, mean 3.08%, median 3.34% for ΔPWV, respectively). Subjects not receiving ACEIs/ARBs showed rates of progression of the MRI plaque measures that were not significantly different between the 2 treatment groups (*P*=0.45, mean 4.32 cm^3^, median 6 cm^3^ for ΔTWV; *P*=0.99, mean 1.88%, median 1.74% for ΔPWV, respectively).

**Table 6.** Effect of ACEI/ARBs on the Changes of the MRI Measures Between Baseline and Follow-up for the 2 Treatment Arms (Wilcoxon Rank Sum Test)

	Placebo (n=19)		Aliskiren (n=18)		
			
	N	Median (IQR)	N	Median (IQR)	*P*
ACEI/ARB therapy					

TWV total	6	−3.01 (−4.49 to −2.71)	11	5.19 (0.10 to 8.56)	**0.02**

TWV thoracic	8	3.03 (2.07 to 4.22)	12	4.38 (3.61 to 5.75)	**0.05**

TWV abdominal	8	−2.22 (−7.21 to 1.57)	11	0.98 (−3.11 to 3.30)	0.36

PWV total	6	−0.06 (−0.88 to 1.27)	11	3.29 (0.91 to 6.36)	**0.03**

PWV thoracic	8	4.2 (1.08 to 5.18)	12	4.83 (3.96 to 6.57)	0.16

PWV abdominal	8	−1.51 (−4.32 to 2.73)	11	0.58 (−3.47 to 5.66)	0.41

No ACEI/ARB therapy					

TWV total	7	0.74 (0.09 to 1.59)	4	6.74 (−0.5 to 13.57)	0.45

TWV thoracic	8	3.41 (2.86 to 5.79)	6	5.33 (1.71 to 11.08)	0.52

TWV abdominal	8	−1.03 (−3.31 to −0.23)	4	0.14 (−2.48 to 3.00)	0.50

PWV total	7	1.33 (0.58 to 1.43)	4	3.07 (−0.004 to 6.92)	0.99

PWV thoracic	8	4.47 (2.85 to 5.51)	6	4.96 (2.87 to 7.74)	0.61

PWV abdominal	8	−1.91 (−3.37 to 0.33)	4	−0.01 (−3.22 to 4.12)	0.62

	ACEI/ARB Therapy (n=22)		NO ACEI/ARB Therapy (n=15)		
Placebo					

TWV total	6	−3.01 (−4.49 to −2.71)	7	0.74 (0.22 to 1.59)	**0.03**

TWV thoracic	8	4.2 (1.08 to 5.18)	8	4.47 (2.85 to 5.51)	0.32

TWV abdominal	8	−1.51 (−4.32 to 2.73)	8	−1.91 (−3.37 to 0.33)	0.87

PWV total	6	−0.06 (−0.88 to 1.27)	7	1.33 (0.62 to 1.4)	0.13

PWV thoracic	8	3.03 (2.07 to 4.22)	8	3.41 (2.86 to 5.79)	0.44

PWV abdominal	8	−2.22 (−7.21 to 1.57)	8	−1.04 (−3.31 to −0.22)	0.79

Aliskiren					

TWV total	11	5.19 (0.26 to 8.04)	4	6.74 (−0.5 to 13.57)	0.75

TWV thoracic	12	5.33 (1.71 to 11.08)	6	4.38 (3.61 to 5.75)	0.96

TWV abdominal	11	0.98 (−3.18 to 2.90)	4	0.14 (−2.48 to 3.00)	0.75

PWV total	11	3.29 (1.13 to 5.78)	4	3.07 (−0.004 to 6.92)	0.94

PWV thoracic	12	4.83 (3.96 to 6.57)	6	4.96 (2.87 to 7.74)	0.89

PWV abdominal	11	0.58 (−3.1 to 4.95)	4	−0.01 (−3.22 to 4.12)	0.94

ACEI indicates angiotensin converting enzyme inhibitor; ARB, angiotensin receptor blockers; PWV, percentage wall volume; TWV, total normalized wall volume.

## Discussion

In this study, we report an unexpected increase in atherosclerosis progression with renin inhibition in high-risk patients who have established cardiovascular disease and receive aliskiren. The majority of the patients in ALPINE had coronary artery disease, with ≍50% of patients additionally demonstrating evidence of left ventricular hypertrophy and/or multiple vascular territory involvement. Mean glomerular filtration rate placed these patients at the cusp of stage 2/3A chronic kidney disease. The patients were normotensive at baseline (<140 mm Hg) on evidence-based therapy but were not at goal for LDL and non–high-density lipoprotein cholesterol despite receiving statin therapy. The inclusion criteria allowed ACEI/ARB drugs, and indeed 60% of patients were taking these drugs. Aldosterone receptor blockers were not used. Peripheral SBP and central SBP were not significantly different between the placebo and aliskiren groups either at baseline or in response to treatment. The lack of significant changes in blood pressure with aliskiren in normotensive patients is similar to a prior randomized trial with Aliskiren.^19^

The rate of progression in total aortic plaque, defined in the study as spanning a distance from the origin of the subclavian artery to the renal arteries, was substantially higher in the aliskiren group. This is despite the fact that the baseline wall volume was higher in the placebo group, a measure that predicts higher rates of progression. The consistency of the finding regardless of the outcome measure (TWV vs PWV) within the constrained duration of follow-up renders the findings all the more significant. The effects of aliskiren on plaque progression appeared to be driven by its effects in patients already receiving ACEI/ARB therapy. Patients not taking ACEI/ARBs who received aliskiren did not demonstrate progression of plaque compared with placebo (Table 6). The interpretation of this finding obviously needs to be taken in context because this was a secondary analysis. The results of this study must be interpreted in the context of a number of important limitations. First, small differences in baseline risk factors may have collectively contributed to differential rates of plaque progression between the 2 groups that may not have had anything to do with an effect of aliskiren. Second, we did not perform qualitative assessment of plaque characteristics to provide insights into whether components of plaque were changing. Third, we did not quantify plaque within regions of the ascending aorta and portions of the arch, which are all well known to have much higher progression rates. Regional differences in plaque progression between groups may have contributed to the results in our study. However, most studies in atherosclerosis that have evaluated therapeutic effects of drugs using MRI-based approaches had the same limitations.^18,20^ Arguably, the use of 3-dimensional approaches spanning a large extent of aorta without gap may have allowed the best possibility to control for some of these effects. To our knowledge, our study represents one of the first studies to examine the change in aortic plaque volume using high-resolution 3-dimensional MRI methods with wall volume calculations akin to intravascular ultrasound studies.^21^ The 3-dimensional coverage of the aorta allows high-resolution assessment of wall volume changes in coregistered images at baseline and the follow-up studies. It has been argued that changes in the aortic wall may represent a superior surrogate for global risk assessment.^13,22^

Interestingly, analysis of plaque progression in the placebo group receiving ACEI/ARB therapy demonstrated significantly smaller progression in TWV at the end of the study, compared with placebo patients who were not taking an RAS antagonist. The rates of MRI progression in the thoracic and abdominal aortas were also distinctly different for the aliskiren and placebo groups. In the placebo group, total aortic TWV and PWV were no different at the end of the study compared with baseline, with only the thoracic segment TWV and PWV increasing significantly. In contrast, the rate of progression in the aliskiren group was substantially increased over the entire aorta compared with baseline, with changes in thoracic segments contributing mostly to these changes. The change from baseline in the PWV measure in the entire aorta was 3.5-fold higher in the aliskiren group compared with the placebo group. The differential rate of progression in the thoracic segment with aliskiren is unclear. It is, however, well known that rates of progression of plaque burden in the arterial tree may depend on the “risk-context,” hemodynamic factors, as well as the unique genetic milieu of the patients that may have collectively interacted with the intervention.^23^ The progression rate in the thoracic segments in our study was not different from that of a previous study that noted greater rates of progression in thoracic segments compared with the abdominal aorta.^24^

How may one, then, explain the results of the study, and are there biologically plausible reasons that would support heightened levels of plaque accrual in patients taking aliskiren? At the outset, it must be noted that all previous studies of aliskiren demonstrated that protective effects in atherosclerosis have been in experimental models, where the therapy was often used in isolation and treatment was administered early in the course of atherosclerosis. These and other differences between animal models and humans have generally resulted in the perception that pharmacological interventions on atherosclerosis in animal models may not be as reproducible in humans.^25^ One notable difference in this study was the increase in leptin levels noted in the aliskerin group. Leptin values were markedly elevated, with most patients meeting criteria for metabolic syndrome based on leptin values, and the levels were consistent with features such as elevated body mass index and the presence of insulin resistance (elevated homeostasis model of assessment–insulin resistance indices).^26^ Leptin has been shown in numerous studies to represent an independent predictor of acute coronary events and stroke after controlling for concomitant risk factors.^27,28^ Leptin values have been previously shown to decrease in response to therapy with ACEI and/or ARB. Statins and calcium channel blockers are for the most part neutral with regard to effects on leptin.^29^ Whether the effect on leptin represents an off-target effect of aliskiren remains to be determined. Of note, these increases occurred despite a trend toward better homeostatic model assessment–insulin resistance indices, a measure of insulin resistance that strongly correlates with leptin levels.^29,30^ Another potential explanation could relate to proximal suppression of angiotensin I synthesis by renin. ACE 2 converts angiotensin I to angiotensin[1–9] or angiotensin II to angiotensin[1–7]. Angiotensin[1–7] has been shown to exert beneficial effects via ligation of the Mas receptor.^31,32^ The use of a renin inhibitor in addition to ACEI or ARB therapy may theoretically lower angiotensin[1–7] synthesis and/or activation of Mas, removing a beneficial cascade.

In conclusion, our study suggests progression of aortic atherosclerosis with renin inhibition in patients with established cardiovascular disease, most of whom were receiving evidence-based therapy, with >50% receiving concomitant ACEI/ARB therapy. In this regard, the ongoing Safety and Efficacy of Aliskiren on the Progression of Atherosclerosis in Coronary Artery Disease Patients (AQUARIUS) trial, which will evaluate the effect of aliskiren on progression of atherosclerosis in patients with established coronary disease on intravascular ultrasound end points, is likely to provide additional information. However the use of ACEI/ARB therapy is proscribed in this trial (http://clinicaltrials.gov/ct2/show/NCT00853827). Our results may have implications for patients using renin inhibitors in conjunction with ACEI and ARB therapy.^33,34^
